# BK Virus Nephropathy in Kidney Transplantation: A State-of-the-Art Review

**DOI:** 10.3390/v14081616

**Published:** 2022-07-25

**Authors:** Sam Kant, Alana Dasgupta, Serena Bagnasco, Daniel C. Brennan

**Affiliations:** 1 Division of Nephrology, Department of Medicine, Johns Hopkins University School of Medicine, Baltimore, MD 21227, USA; dbrenna4@jhmi.edu; 2 Comprehensive Transplant Center, Johns Hopkins University School of Medicine, Baltimore, MD 21227, USA; 3 Department of Pathology, Johns Hopkins University School of Medicine, Baltimore, MD 21227, USA; adasgup3@jhu.edu (A.D.); sbagnas1@jhmi.edu (S.B.)

**Keywords:** BK virus, kidney transplantation, viremia, nephropathy, kidney injury

## Abstract

BK virus maintains a latent infection that is ubiquitous in humans. It has a propensity for reactivation in the setting of a dysfunctional cellular immune response and is frequently encountered in kidney transplant recipients. Screening for the virus has been effective in preventing progression to nephropathy and graft loss. However, it can be a diagnostic and therapeutic challenge. In this in-depth state-of-the-art review, we will discuss the history of the virus, virology, epidemiology, cellular response, pathogenesis, methods of screening and diagnosis, evidence-based treatment strategies, and upcoming therapeutics, along with the issue of re-transplantation in patients.

## 1. Introduction

BK virus-associated nephropathy (BKVAN) is an important cause of graft loss in kidney transplant recipients. The successful advent of increasingly efficacious immunosuppression has been accompanied by high rates of BK viremia (BKV) in up to 30% of kidney transplant recipients [1]. Since its discovery in 1971, an effective prophylaxis or therapy is yet to be devised, with unmitigated disease frequently resulting in allograft loss. This review provides an extensive overview of viral epidemiology, pathogenesis, screening, and diagnostic methods. In addition, we discuss clinical manifestations and recommended treatment strategies. 

## 2. History of the BK Virus

BK virus was first discovered in a kidney transplant recipient who presented with a ureteral stricture in 1971 [2]. However, it was only in 1993 that the first definitive biopsy-proven case of BKVAN was described [2,3]. It is a matter of debate whether the increasing incidence of BK viremia over the subsequent years was as a result of the increasing availability of reliable testing methods versus a consequence of more potent immunosuppression regimes. During the period of its early description, BKVAN frequently resulted in graft loss with rates of 50–100% reported [3]. The ensuing increasing recognition and nuanced management have now resulted in a reduction in associated graft loss to under 15% within the last 2 decades [4]. 

## 3. Virology

BK virus is a small, non-enveloped, icosahedral, closed circular, double-stranded DNA virus and member of the Polyomaviridae family [5]. The genome of the virus consists of three regions—the early coding region of the large T and small t antigens (large and small tumor antigen), the non-coding control region, and the late coding region. 

The T antigen has a propensity to bind to p53 and protein Rb, resulting in the commencement of its cell cycle in host cells and subsequent persistent infection. The non-coding control regions are a significant contributor to the pathogenesis of the virus since it contains the origin of replication and enhancer elements that can modulate transcription. Mutations in the non-coding control regions result in permit replication in other cell types (permissivity), cell tropism, and altered rates of replication [6,7,8]. These mutations correlate with high BK virus loads in kidney transplant recipients with clinically significant viral replication [7].

The late-coding region codes for the agnoprotein and viral capsid proteins (VP-1, VP-2, and VP-3). The agnoprotein is responsible for the assembly of viral capsids and the release of virion from cells [9]. VP-1 is the major structural protein that engages with cellular receptors and has significant genetic heterogeneity—this recognition has led to the classification of viral genotypes I to VI. Serotype I has been the predominant genotype and is implicated in most clinically significant viral diseases. It is notable that the generation of antibodies against one serotype does not result in durable protection against other types [10,11]. The VP-2 and VP-3 act as nuclear location signals, thereby aiding the navigation of virions to the host cell nuclei. In addition, VP-3 leads to activation of the adenosine diphosphate-ribose polymerase, resulting in depletion of adenosine triphosphate (ATP) and cell death [3].

## 4. Epidemiology

BK virus infection could be considered ubiquitous in the general population, with seroprevalence rates of over 90% by 4 years of age [12,13,14]. The primary routes for transmission of the virus are from mucosal contact including the oral, gastrointestinal, and respiratory tract. After a primary viremia, the BK virus establishes refuge in the kidney and uroepithelial cells resulting in lifelong latent/persistent infection. 

Since cellular immunity is most suppressed in the first post-transplant year as a result of induction therapy, viral replication can frequently ensue during this period. Clinically significant infection occurs in kidney transplant recipients via reactivation of latent infection or transmission of new infection from the donor kidney. The infection occurs in the following chronological stages—viruria, viremia, and allograft nephropathy [15,16]. Viruria and viremia are detected in approximately 30% and 12% of kidney transplant recipients, respectively [17,18]. After the onset of viruria, nearly 50% of kidney transplant recipients develop viremia during a period of 2–6 weeks, with a similar proportion of viremic patients developing BKVAN in the aforementioned time period [18,19,20,21,22]. This correlates with the clinical observation that urine BK viral loads >8 log_10_ c/mL predict the onset of viremia, while plasma BK viral loads >4 log_10_ c/mL are associated with higher rates of biopsy-proven BKVAN and loads peaking above 6 log_10_ c/mL are predictive of extensive BKVN pathology measured by SV40 immunohistochemistry and associated inflammatory infiltrates [20,23,24,25,26,27,28]. Based on the most recent registry data, 1–10% of kidney transplant recipients develop BKVAN [20,29,30,31,32]. 

Viral replication is associated with the following risk factors:

1. Intensity of immunosuppression: This is considered as the most significant factor associated with BK viral replication. This is based on the finding that the incidence of BK viremia is highest in the early post-transplant period given the magnitude of immunosuppression. Some but not all studies have demonstrated that tacrolimus may portend a higher risk of BK virus infection than cyclosporine, but this is confounded by the fact that cyclosporine inhibits enterohepatic recirculation of MMF and results in lower mycophenolic acid area under the curve [31,33,34,35], while mammalian target of rapamycin (mTOR) inhibitors may be associated with lower risk by virtue of being less immunosuppressive than tacrolimus or cyclosporine [36,37,38]. It is pertinent to note the presence of BK viremia reflects a higher level of immunosuppression and its occurrence with or without BKVAN is in the setting of all maintenance immunosuppressive agents/combinations [18,39,40,41,42,43,44,45].
2. Recipient characteristics: older age [33,46], diabetes [1], and specific HLA-C alleles [47]
3. The donor–recipient interface: The high-risk serostatus of donor positive and recipient negative for BK virus [48,49], ABO incompatibility, HLA mismatch [50], delayed graft function [15], rejection or ischemia of the transplanted kidney [51], and ureteral stent placement [15].
4. Donor-related factors: reduced immune response to BK virus [48,52] and BK viruria prior to transplant [19,53,54].

Recipient HLA-B51 positivity [55] and the presence of polycystic kidney disease [56] have been shown to be protective factors against the development of BKVAN. HLA-B51 positivity is associated with the presence of highly immunogenic cytotoxic T cells, which may explain the fivefold reduction in the occurrence of BKVAN in these patients [55].

## 5. Cellular Immune Response and Pathogenesis 

The mitigation and clearance of BK viremia are dependent on a robust cellular immune response—with both CD4 and CD8 cells playing a crucial role in this process [57]. The BK capsid proteins, large T antigen, and non-structural proteins elicit T cell responses, which can be quantified with the use of enzyme-linked immunosorbent spot (ELISPOT) and tetramer staining. 

The presence of ELISPOT measured IFN-gamma activity, indicative of a BK-directed cellular immune response, is associated with the resolution of BKVAN [58]. The shorter time interval (<1 month) to develop anti-BK T cell response correlates with the clearance of viremia, while patients who develop BKVAN required a median period of 5 months to develop cellular immunity against the virus [59]. Additionally, vigorous CD8-based cellular responses correlated with lower BK viral loads in blood and urine, while high viral loads and the continued presence of the virus were associated with a weak response. BK-directed cytokine signatures from CD4 cells have demonstrated similar results [60], providing further evidence that a concerted effort from components of the cellular immune system is vital for tempering the virus. Various studies are currently being conducted to assess if these assays could be utilized to predict the clearance of the BK virus and identify patients at high risk for progression of the virus-associated disease. 

The virus maintains persistent infection after initial childhood infection and maintains refuge in kidney epithelium, mostly in the parietal epithelium of the Bowman’s capsule, renal tubular epithelium, and transitional epithelium [61,62,63]. Other sites of latent infection include prostate, testes, seminiferous tubules, cervix, vulva, and hematolymphoid tissues (peripheral blood mononuclear cells, and tonsils). This latent infection can become active with reduced potency of cellular immunity after the introduction of immunosuppression. Damage to tubular epithelium results from the ensuing viral replication and its cytopathic effect, with continued inflammation leading to the activation of pro-fibrotic pathways (transforming growth factor *β*, matrix metalloproteinase-2, matrix metalloproteinase-9, and matrix collagens) [64]. BKVAN is also associated with increased expression of various major groups of messenger RNAs (mRNAs), including CD8, perforin, interferon-*γ*, and CXCR3. These mRNAs are also expressed in T cell-mediated rejection (TCMR) and could explain common clinical and pathological features in BKVAN and TCMR with associated difficulty in distinguishing the two entities [65]. The final stage of destruction is characterized by interstitial fibrosis and tubular atrophy, with associated progressive nephron loss [66].

## 6. Clinical Manifestations

Most clinically significant infections associated with the BK virus lack any systemic symptoms. The classic sequence of infections in kidney transplant recipients is viruria, viremia, and BKVAN. The most common and earliest manifestation of BKV is viruria occurring in up to 50% of patients in the first year of transplantation, with most cases not progressing to viremia [17,18]. The checking of urine BK viral loads is not standard practice despite it being a sensitive marker for progression to BKVAN [67]. This is because of the non-specific nature of viruria occurring without any risk of progression in pregnant women, older patients, and those with compromised cellular immunity (other than transplant recipients) [46,68]. 

The presence of sustained viruria may progress to viremia, which is asymptomatic initially. Viremia is present in 10–30 percent of recipients in the first six months post-transplantation and in 5–10 percent of recipients thereafter [67,69]. Viremia is a better predictor of progression to BKVAN in comparison to viruria [17,69]. 

BKVAN usually occurs after a period of sustained progressively worsening viremia, manifesting as a decline in renal function with or without urinary abnormalities. The vast majority of BKVAN occurs within the first post-transplant year given attenuated cellular immunity, with the first 2–6 months being periods of highest incidence [70]. Other manifestations of the BK virus include ureteral stenosis and hemorrhagic cystitis—albeit rare in kidney transplant recipients and mostly seen in patients with hematopoietic stem cell transplants. There are reports of a possible link between the BK virus and genitourinary (GU) malignancies, especially given its protracted infection in epithelia of the GU tract. A causative role in malignancies in humans is controversial given conflicting results regarding the presence of BKV sequences and/or proteins in various tumor types, with animal and in-vitro studies demonstrating BK-induced oncogenesis and cell transformation [71,72,73]. There are accumulating reports that there may be an association of the BK virus with the development of urothelial malignancies in transplant recipients [74,75]; however, a possibility of confounding exists since patients who develop BKVAN have lowered cellular immunity, and as a result of reduced tumor surveillance are at risk of development of malignancies. 

## 7. Screening and Diagnosis

The high incidence of BK viremia in the first post-transplant year has led to the development of standard screening protocols by transplant centers. Given the low specificity of urine BK viral loads, and higher positive predictive value of plasma BK levels, screening for BK viremia is the preferred method utilized in these protocols [76].

Fastidious screening and preemptive reduction in immunosuppression for established BK viremia have been demonstrated to mitigate progression to BKVAN [34,77,78]. The Kidney Disease: Improving Global Outcomes (KDIGO) and American Society of Transplantation Infectious Diseases Community of Practice (AST-IDCOP) guidelines recommend monthly screening for the first 6 months post-transplantation and then every 3 months for the next 18 months [1,79].

### 7.1. Urine BK PCR

Urine BK PCR is not a recommended screening test given issues related to specificity and cost—if positive, it always requires confirmation with plasma PCR and nearly fifty percent of patients with viruria will not develop viremia [80]. 

### 7.2. Plasma BK PCR

BK viral loads are measured by polymerase chain reaction (PCR)—a fluorescent probe BK-specific sequence, and the number of amplicons produced is compared with a standard curve generated with serial dilutions of a known concentration of BK DNA [57]. Assay results are influenced by variations in DNA extraction techniques, sample type/source, primer and probe sequences, and BK strain DNA used for standard-curve BK virus genotype variance and discordant BK viremia PCR assay results. Given that inter-assay variability makes the accurate measurement of viral loads difficult, the World Health Organization (WHO) addressed this by establishing an international standard to standardize viral load values among different laboratory assays when results are expressed as international units/mL [81]. While there has been improvement in the reporting of BK PCR values since the introduction of this international standard in 2016, there continues to be variability among laboratories attributed to PCR primer design DNA extraction techniques and amplicon size [82,83,84].

The genotypes of the BK virus detected by the PCR assays warrant special discussion. The genotype I (Dunlop) strain is currently utilized as the reference sequence against which primers and probes are designed for various assays [85]. However, there is significant discordance among various assays with primer or probe mismatch due to subtype-associated polymorphisms, primarily among subtype III and IV isolates [86]. Moreover, BK PCR assays can be four times less sensitive for variant strains when using genotype I as a reference (limit of detection of 10,000 copies/µL for the variant strain compared with 10 copies/µL for genotype I) [85]. This could lead to the non-detection of rarer genotypes, which are being recognized to be more cytopathic and more frequently associated with BKVAN. Therefore, rare genotypes should be considered in the event that BKVAN is co-existent with lower viral loads. 

### 7.3. Urine Cytology

**Figure 1:**
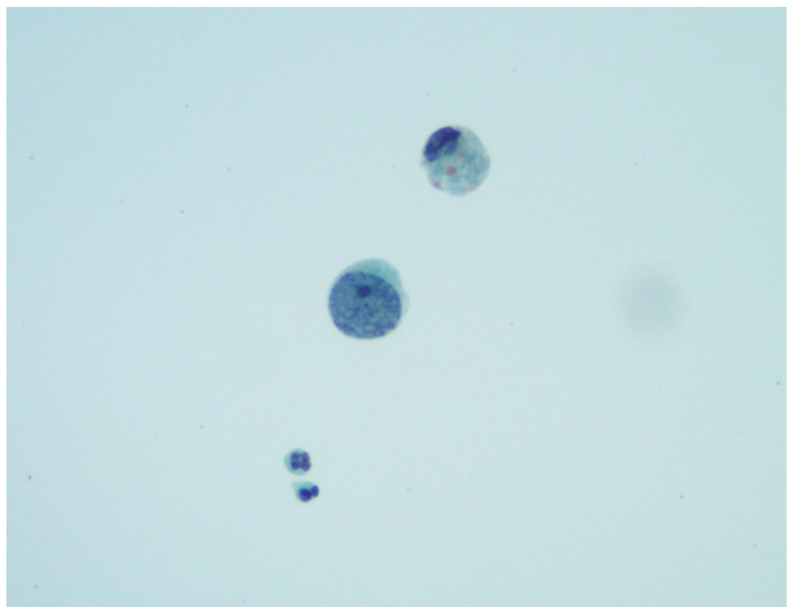
A “decoy cell” with an enlarged nucleus and clumped chromatin, mimicking high-grade urothelial atypia. PAP smear 600×. Courtesy of Zareema Mangaru, DO.

The characteristic BK virus-infected cells that present on cytologic examination of urine are called ′decoy cells′ due to their similarity to renal carcinoma cells. These are tubular epithelial or urothelial cells with ground-glass nuclear inclusions surrounded by a condensed rim of chromatin (Figure 1). They may also exhibit “owl eye” inclusions, multinucleation, or clumped chromatin. Although decoy cells are a marker of PV replication, they do not necessarily indicate PVN. Some studies have shown high false-positive rates and low positive predictive values when attempting to use the presence of decoy cells to screen for PVN in transplant patients [27,87,88]. However, some of these studies also found the absence of decoy cells in urine cytology screens had high negative predictive values for PVN. Urine samples may also be screened for the presence of cast-like PV aggregates, called polyomavirus-Haufen, via negative staining electron microscopy [89]. The presence or absence of PV-Haufen has extremely high positive and negative predictive values for BK nephropathy, respectively. Additionally, the amount of PV-Haufen shed correlates well with disease severity [90].

### 7.4. Donor-Derived Cell-Free DNA (dd-cfDNA)

A recent study evaluated the association of dd-cfDNA with plasma BK viral loads and biopsy findings to determine if dd-cfDNA can distinguish asymptomatic BKV from BKVAN [91]. It demonstrated that higher dd-cfDNA levels were associated with higher BK viral loads, biopsy-diagnosed BVAN, as well histologic changes meeting Banff criteria for T-cell-mediated rejection. These preliminary findings show that dd-cfDNA may be a useful noninvasive test to assess for progression of BKV to BKVAN [92,93].

### 7.5. Transplant Kidney Biopsy

Kidney allograft biopsy continues to be the gold standard for the diagnosis of BVAN. It aids, not only in diagnosis, but also in assessing the severity of viral involvement and the presence of other ongoing pathologies. Since BK is tropic for the medulla, it is necessary that the biopsy core has medulla present to decrease the likelihood of a sampling error (see below).

The following pathologic features should be present for a definitive diagnosis of BKVAN [39,94,95]:

1. Characteristic cytopathic changes (described further in *Histology* below).
2. Positive immunohistochemistry tests using antibodies directed specifically against BKV or against the cross-reacting SV40 large T antigen. Positive SV40 staining is useful as it is associated with a specificity of almost 100 percent for polyomavirus nephropathy (PVN); although, it does not distinguish between BKV and JC virus (JCV).

A presumptive diagnosis of BKVAN is considered in the setting of plasma BK viral load ≥10,000 copies/mL. Given that BK mostly affects the medulla and is associated with focal disease, diagnosis via a kidney biopsy is estimated to be missed in nearly 30% of cases [28,95]. If the initial biopsy is not confirmatory for BKVAN, a repeat biopsy is recommended if clinical suspicions remain.

### 7.6. Histology

**Figure 2:**
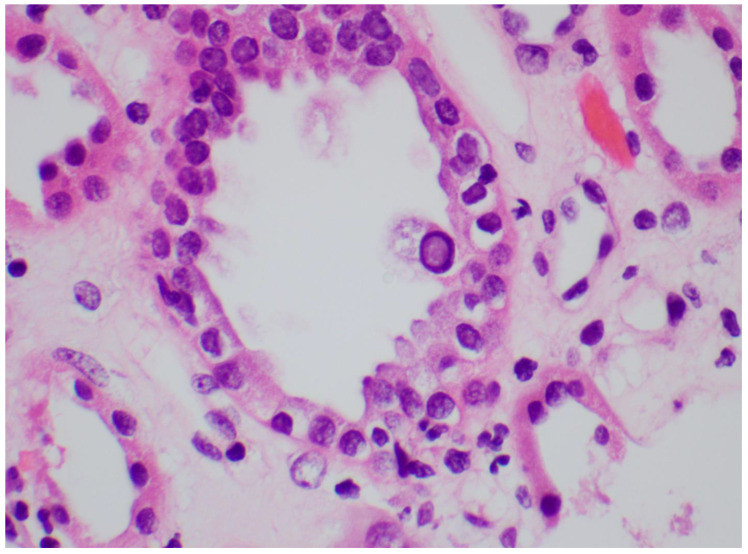
A tubular epithelial cell with a “ground glass” nuclear inclusion. H&E 600×.

**Figure 3:**
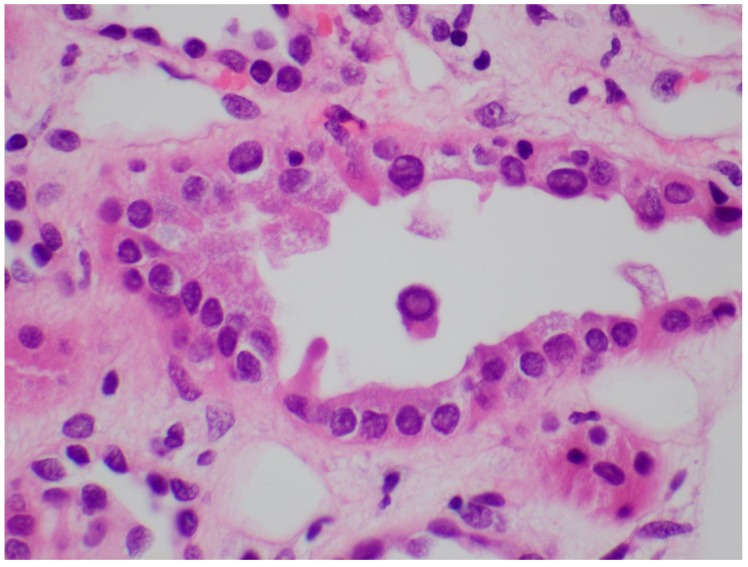
A tubular lumen containing sloughed epithelial cells with a viral intranuclear inclusion. H&E 600×.

**Figure 4:**
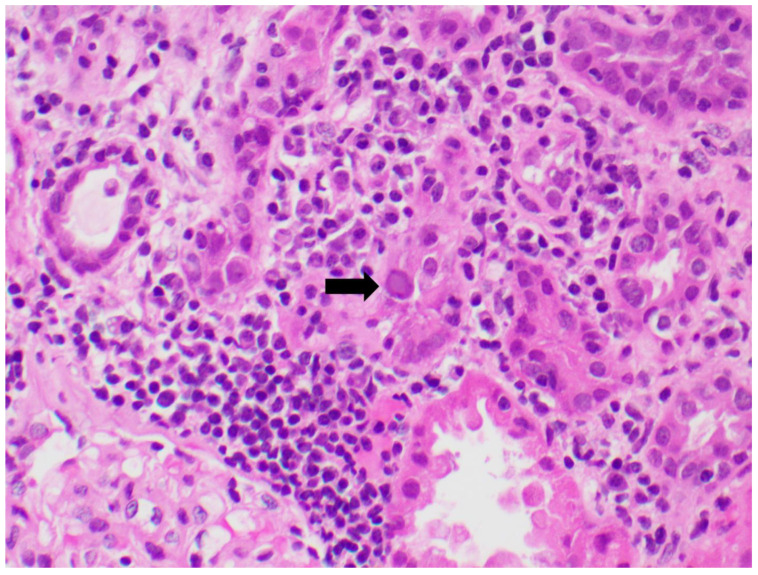
Lymphoplasmacytic interstitial inflammation surrounding a tubule containing an epithelial cell with a viral inclusion (arrow). H&E 400×.

Identifying the histologic features of polyoma virus infection on renal biopsy is currently the gold standard for the diagnosis of “definitive” BKVAN. In the genitourinary tract, these viruses target urothelial and renal tubular cells, resulting in virion production and subsequent cellular destruction [96]. The histologic findings of this process include tubular epithelial cells with enlarged, hyperchromatic nuclei and “ground glass” intranuclear inclusions (Figure 2) [97]. Viral cytopathic changes may also include granular nuclear inclusions and “clumps” of intranuclear virion particles. In the early stages of infection, only rare tubular cells with viral cytopathic changes may be seen, usually in the distal nephron or medulla. Eventually, these cells lyse and slough from the basement membrane into the tubular lumens (Figure 3). As the infection progresses, tubulitis and interstitial inflammation with a prominent plasma cell component may be seen (Figure 4). More proximal portions of the nephron, including the parietal epithelial cells lining Bowman′s capsule, may also become involved. The tubular cell injury, tubulitis, and interstitial inflammation may result in tubular atrophy and interstitial fibrosis.

**Figure 5:**
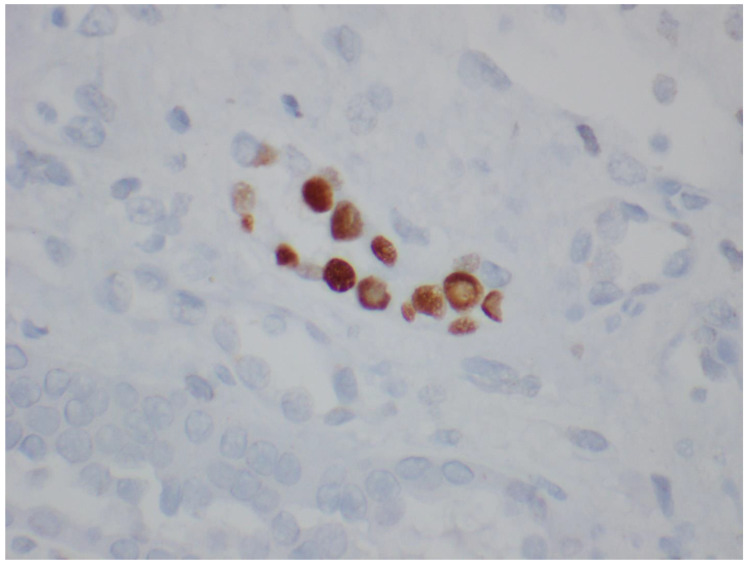
SV40 IHC staining highlighting infected tubular epithelial cells (600×).

**Figure 6:**
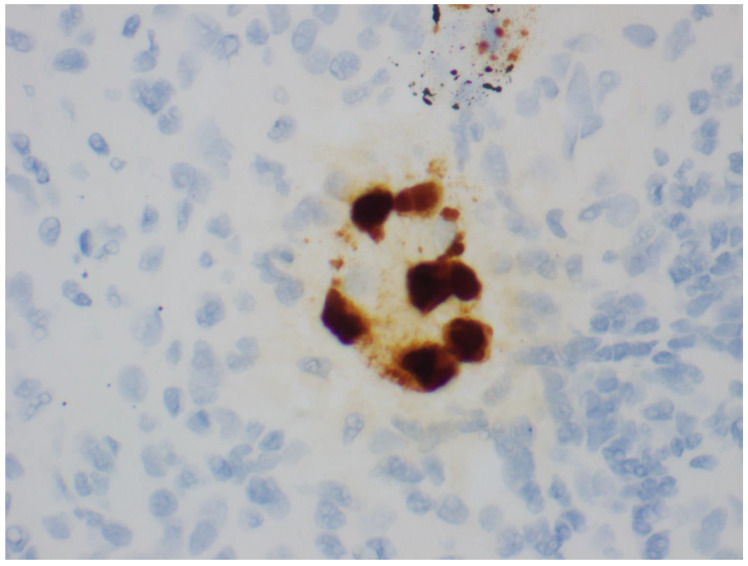
In situ hybridization for BK virus RNA (600×).

Histologic confirmation of the presence of polyoma virus can prove difficult. The random and focal nature of the infection can result in false negatives if uninvolved parenchyma is sampled [28]. To maximize the likelihood of identifying diagnostic features, two biopsy cores containing renal cortex and medulla are recommended [98,99]. Additionally, PV cytopathic changes can be focal, subtle, and potentially overlooked, especially in the early stages. To aid in the detection of PV within biopsies, ancillary tests such as immunohistochemical (IHC) stains or in situ hybridization (ISH) can be performed. Currently, it is recommended that the Simian Virus 40 (SV40) IHC stain be performed on all transplant biopsies where PVN is suspected clinically, but no definitive features of PVN are seen [99]. The SV40 IHC stain detects the large T antigen expressed by all polyoma viruses pathogenic in humans (SV, JC, and BK). This stain can highlight cells in the early stages of infection, before viral cytopathic changes may be detectable on routine stains (Figure 5 and Figure 6). This stain may also help differentiate PVN from other viral nephropathies seen in immunocompromised patients, such as adenovirus infection. Quantitative PCR for PV can also be performed on tissue samples [100]. However, given the high frequency of latent PV virus infection, interpretation of these results requires caution. Finally, PV can be identified on electron microscopy by the presence of 40 nm paracrystalline viral particles within the nuclei of tubular cells. 

Adding further difficulty to the diagnosis of PVN are the histologic similarities it shares with acute rejection (AR). Because the treatment for these two entities is usually diametrically opposed, arriving at the correct diagnosis is of exceptional clinical importance. In rare cases, AR and PVN can co-occur, creating a treatment dilemma [101]. In both cases, tubular injury, tubulitis, and interstitial inflammation are key histologic findings [102].The presence of these findings in the absence of morphologic or immunohistochemical evidence of PV infection should warrant consideration of acute cellular rejection. A plasma cell-rich infiltrate or injury found primarily in the medulla should raise concern for PVN. A review of deeper levels or ancillary stains to identify evidence of viral infection should be considered in these situations. However, plasma cell-rich infiltrates are not PV specific and can be seen in TCMR [103]. With rare exceptions, features of vascular injury such as endarteritis, arterial fibrinoid necrosis, glomerulitis, or peritubular capillary C4d staining are more consistent with acute rejection than PVN [101,102]. In cases of histologic overlap, a diagnosis of concomitant PVN and AR should be considered. In these instances, the histologic findings should be correlated with the clinical history and results of additional laboratory studies, such as BK viral load and the presence of donor-specific antibodies (DSA). A review of the biopsy and discussions with the clinical team may also prove insightful. 

Several grading systems have been proposed for the evaluation and reporting of PKN, including those developed by the University of Maryland, the American Society of Transplantation, and the Banff Working Group [28,99]. These systems use features such as viral cytopathic effect, injury, and atrophy to classify cases. The Banff Working Group on Polyomavirus Nephropathy Classification System is a three-tier scoring approach that incorporates the extent of morphologic evidence of PV infection and interstitial fibrosis to classify samples (Table 1) [99]. As per this system, histologic classes of definitive PVN are defined by the morphologic degree of intrarenal pvl (polyomavirus replication/load level) and Banff ci scores (interstitial fibrosis). The scoring of pvl is on the basis of the extent of virally induced tubular changes, while a tubule with intranuclear viral inclusion bodies (type 1 or 2) and/or a positive IHC reaction for SV40-T antigen in one or more cells per tubular cross-section is considered “a positive tubule”. The overall percentage of positive tubular cross-sections is estimated in the entire biopsy sample (all available cores, cortex, and medulla). The consistent use of a grading system may reduce the degree of intra and inter-observer variability and facilitate communication between the pathologist and clinical team.

## 8. Treatment

A reduction in the intensity of immunosuppression is the overarching principle for the treatment of BK viremia and BKVAN. There is no therapeutic agent available to treat this virus-associated disease, with many agents lacking conclusive efficacy in the reduction in viral loads. Multiple protocols have been developed for a reduction in immunosuppression, albeit trials are yet to be conducted to compare their effectiveness. 

The following is a stepwise approach for the reduction in immunosuppression in the setting of BK viremia and BKVAN based on trials in adult and pediatric kidney transplant recipients [40,104]:

1. Reduce dose of antimetabolite by half while continuing on the same doses of calcineurin inhibitor and/or prednisone. It is imperative to monitor serum creatinine and serial plasma BK PCR levels from the same laboratory (to reduce inter-assay variability) every 2 weeks in the interim.
2. If viral loads continue to be at similar levels or increase, proceed with complete cessation of anti-metabolite.
3. The next step is to reduce calcineurin-inhibitor trough goals if viral loads do not reduce over 4 weeks despite cessation of anti-metabolite (4–6 ng/mL for tacrolimus and 50–100 ng/L for cyclosporine).

It is pertinent to note that based on the 5-year follow-up data of patients with BK viremia and BKVAN, kidney transplant recipients maintained on tacrolimus-based regimes had lower rates of rejection and a higher estimated glomerular filtration rate (eGFR) [40]. 

Other adjunctive therapies utilized to treat BK virus infection include quinolones, cidofovir, leflunomide, and intravenous immunoglobulin (IVIG). A meta-analysis has demonstrated that there is no difference in graft outcomes when the strategy of reduction in immunosuppression is compared with a combination of immunosuppression with leflunomide or cidofovir [105]. Intravenous immunoglobulin is probably the only viable adjunctive therapy, while the use of the other aforementioned agents is not recommended. The discussion of this aspect is presented below:

1. IVIG: It is utilized in the setting of non-response to a maximal reduction in immunosuppression (balancing with risk of rejection). The rationale for use is the presence of BK-neutralizing antibodies in IVIG preparations. Data from five observational studies have demonstrated a reduction in BK viral loads; however, other anti-viral agents were administered at the same time as well [106,107,108,109,110].
2. Quinolones: Despite demonstrating anti-viral properties in vitro, randomized trials failed to show efficacy as prophylaxis in the immediate post-transplant period or treatment for BK viremia [111,112,113]. In the levofloxacin prophylaxis trial, a higher incidence of resistant bacterial infection was seen in the quinolone group [112].
3. Cidofovir: A nucleotide analog of cytosine has demonstrated activity against Polyomaviridae in-vitro [114]. Studies have shown no benefit with cidofovir use, notwithstanding that a significant risk of kidney dysfunction was noted [115,116]. Cidofovir has already been shown to be associated with proteinuria, proximal tubular dysfunction, and kidney disease [114,117].
4. Leflunomide: A prodrug that converts to an active metabolite, A77 1726, which has demonstrated both immunosuppressive and anti-viral properties [118,119]. While there was initial enthusiasm for its use in BK virus infection based on a case series, a pharmacodynamic and prospective open-label study showed no benefit [119,120,121]. Another metabolite, FK778, did not demonstrate efficacy in a phase 2, proof-of-concept, randomized, open-label, parallel-group, 6-month study in kidney transplant patients when compared with a reduction in immunosuppression [122].

### Upcoming Therapeutic Trials

Given the lack of therapies available for the treatment of BK infection, the two following trials involving modified T cells and monoclonal antibodies are currently underway:

1. A randomized, double-blind, placebo-controlled study to assess the safety, pharmacokinetics, and efficacy of MAU868—a human monoclonal antibody (IgG1) that binds the viral capsid protein, VP1, which is responsible for binding to the surface of host cells (ClinicalTrials.gov identifier: NCT04294472).
2. A phase 2 multicenter, randomized, double-blind, study of the safety, tolerability, and effectiveness of adoptively transferred posoleuccel (ALVR105) multivirus-specific T Cells in kidney transplant recipients with either high or low levels of BK viremia (ClinicalTrials.gov identifier: NCT04605484).

## 9. Kidney Re-Transplantation

Patients with graft loss due to BKVAN should be considered for re-transplantation given its extensive evidence of success [123,124,125,126,127]. Failed transplant or native nephrectomy is not recommended given the lack of evidence-based guidelines to substantiate this practice and confirmation of viral clearance should be made prior to transplantation. Consideration for lower immunosuppression should be balanced with the risk of rejection. 

Allograft survival in patients who receive re-transplantation is 98% and 94% at 1 and 3 years, respectively [128]. In comparison to re-transplanted patients for graft failure from other causes, five-year death-censored graft survival rates were 91% for the BKVAN group and 84% for the non-BKVAN group. Additionally, there was no significant difference in the rates of acute rejection or patient survival at one year [123]. 

## 10. Conclusions

BK virus infection continues to be one of the most common clinical issues encountered by transplant providers. Heightened surveillance protocols have led to expedient detection and have mitigated severe disease. It also can be a conundrum from a diagnostic and therapeutic standpoint. It can be associated with histologic features akin to rejection, with a reduction in immunosuppression being the only viable treatment strategy, which may itself culminate in rejection. In addition, there is no anti-viral currently known to be of benefit in the clearance of the virus. There is, however, promise that novel therapeutics may bring efficacy that continues to elude the transplant community. 

## Figures and Tables

**Table 1:** Histologic classification system of PVN for the Banff Working Group Classification of Definitive Polyomavirus Nephropathy.

Biopsy-Proven PVN Class 1		Biopsy-Proven PVN Class 2		Biopsy-Proven PVN Class 3	
pvl	Banff Ci Score	Pvl	Banff Ci Score	Pvl	Banff Ci Score
1	0–1	1	2–3	-	-
-	-	2	0–3	-	-
-	-	3	0–1	3	2–3

The pvl score is calculated based on the extent of virally induced changes identified on H&E or via SV40 IHC staining. Scores for pvl are calculated as follows: pvl1: ≤1% of all tubules/ducts with viral replication; pvl2: >1 to ≤10% of all tubules/ducts with viral replication; pvl3: >10% of all tubules/ducts with viral replication. The ci score is calculated using the Banff Classification of Renal Allograft Pathology. Ci—interstitial fibrosis; pvl: polyomavirus replication/load level (pvl); PVN: polyoma virus nephropathy.
